# Inhibition of the Notch1 pathway induces peripartum cardiomyopathy

**DOI:** 10.1111/jcmm.15423

**Published:** 2020-06-11

**Authors:** Rong‐rong Zhu, Qian Chen, Zhi‐bo Liu, Han‐guang Ruan, Qi‐cai Wu, Xue‐liang Zhou

**Affiliations:** ^1^ Department of Obstetrics and Gynecology High‐Tech Hospital The First Affiliated Hospital Nanchang University Nanchang China; ^2^ Department of Cardiology the Second Medical Center, Chinese PLA General Hospital, National Clinical Research Center for Geriatric Diseases Beijing China; ^3^ Department of Cardiovascular Surgery The First Affiliated Hospital Nanchang University Nanchang China; ^4^ Department of Medical Oncology The Third Hospital of Nanchang City Nanchang University Nanchang China

**Keywords:** Cathepsin D, Notch1, PPCM, PRL, sFlt1

## Abstract

Increased expression and activity of cardiac and circulating cathepsin D and soluble fms‐like tyrosine kinase‐1 (sFlt‐1) have been demonstrated to induce and promote peripartum cardiomyopathy (PPCM) via promoting cleavage of 23‐kD prolactin (PRL) to 16‐kD PRL and neutralizing vascular endothelial growth factor (VEGF), respectively. We hypothesized that activation of Hes1 is proposed to suppress cathepsin D via activating Stat3, leading to alleviated development of PPCM. In the present study, we aimed to investigate the role of Notch1/Hes1 pathway in PPCM. Pregnant mice between prenatal 3 days and postpartum 3 weeks were fed with LY‐411575 (a notch inhibitor, 10 mg/kg/d). Ventricular function and pathology were evaluated by echocardiography and histological analysis. Western blotting analysis was used to examine the expression at the protein level. The results found that inhibition of Notch1 significantly promoted postpartum ventricular dilatation, myocardial hypertrophy and myocardial interstitial fibrosis and suppressed myocardial angiogenesis. Western blotting analysis showed that inhibition of Notch1 markedly increased cathepsin D and sFlt‐1, reduced Hes1, phosphorylated Stat3 (p‐Stat3), VEGFA and PDGFB, and promoted cleavage of 23k‐D PRL to 16‐kD PRL. Collectively, inhibition of Notch1/Hes1 pathway induced and promoted PPCM via increasing the expressions of cathepsin D and sFlt‐1. Notch1/Hes1 was a promising target for prevention and therapeutic regimen of PPCM.

## 1 INTRODUCTION

Peripartum cardiomyopathy (PPCM) remains a frequently fatal disease of unknown aetiology, and its pathogenesis remains largely unexplored. Increasing evidence supports that cardiac angiogenic imbalance contributes to PPCM, which is caused by excessive anti‐angiogenic factors and reduced angiogenic factors.^1^ Mechanistically, cathepsin D–cleaved 16‐kD prolactin (PRL), an anti‐angiogenic and pro‐apoptotic form of 23‐kD PRL, suppresses cardiac angiogenesis via inhibiting proliferation and migration of vascular endothelial cells and promoting their apoptosis, leading to PPCM.^2^ Additionally, elevated soluble fms‐like tyrosine kinase‐1 (sFlt‐1) has also been demonstrated to impair cardiac capillary network through inhibiting pro‐angiogenic vascular endothelial growth factor (VEGF) and placental growth factor (PIGF) activities.^3^ The combination of bromocriptine (PRL inhibitor) and recombinant VEGF is a curative option for PPCM.^1^, ^4^

As a canonical target gene of Notch pathway, Hes1 has been found to regulate angiogenesis.^5^, ^6^ Our previous work has suggested that Hes1 is able to protect ischaemic myocardium via mediating the phosphorylation of Stat3.^7^ Reportedly, Stat3 modulates proliferation, differentiation, survival, oxidative stress and/or metabolism in cardiomyocytes, fibroblasts, endothelial cells, progenitor cells and various inflammatory cells.^8^ Indeed, Hilfiker‐Kleiner ^2^ and colleagues have found that activated Stat3 can inhibit cathepsin D, which then suppresses PPCM development. Furthermore, our team has also found that Notch1 can promote VEGF‐mediated cardiac angiogenesis in ischaemic regions ^9^ and inhibit myocardial fibrosis.^10^, ^11^ Bioinformatic analysis shows that there are multiple Hes1 binding sites in the promoter region of cathepsin D and sFlt‐1, indicating a potential role of Hes1 in PPCM.

We hypothesized that inhibition of Notch1/Hes1 induced and promoted PPCM via increasing cathepsin D and sFlt‐1. In the present study, we used LY‐411575 (γ‐secretase inhibitor) to suppress Notch1 pathway and decrease Hes1 expression ^12^ to explore the potential role of Notch1/Hes1 in PPCM.

## 2 MATERIALS AND METHODS

### 2.1 Animal experiments

Female C57BL/6J mice (6‐8 weeks of age) were purchased from Slaccas Co., Ltd. All animal studies were performed at Experimental Animal Center of Nanchang University in accordance with the Guideline of US National Institutes of Health (NIH), and animal‐related protocols were approved by the Institutional Committee for Use and Care of Laboratory Animals of Nanchang University. The mice with PPCM during pregnancy and breastfeeding were assigned into the peripartum group, while the nulliparous mice were used as the control group. Mice were administered by gavage with LY‐411575 (10 mg/kg/d, diluted in 0.4% methylcellulose) daily starting 3 days before delivery until 3 weeks after delivery. The blank mice were dosed with 0.4% methylcellulose vehicle. The PPCM phenotype was verified as previously described.^13^ At the end of the dosing period, mice were sacrificed by CO_2_ asphyxiation, total blood was collected and centrifuged at 1500 g for 10 minutes at room temperature to obtain serum. Serum samples were stored at −80°C until analysis. The heart tissues were surgically isolated for further analysis.

### 2.2 Reagents

LY‐411575 was purchased from Selleck (Cat. S2714). BCA protein assay kit was obtained from Pierce. The Cathepsin D (ab239420) ELISA kit was purchased from Abcam. The sFlt1 (DY471) ELISA kit was purchased from R&D System. Rabbit anti‐PRL antibody (ab110642), rabbit anti‐VEGFA antibody (ab52917), rabbit anti‐PDGFB antibody (ab178409) and rabbit anti‐cathepsin D (ab75852) antibody were supplied by Abcam. Mouse anti‐BFGF (5414), anti‐phos‐STAT3 (9145), mouse anti‐STAT3 (9139), rabbit anti‐N1ICD (4147) and rabbit anti‐N1ICD (11 988) antibodies were provided by Cell Signaling Technology. Goat anti‐rabbit IgG, goat antimouse IgG and rabbit anti‐HRP‐GAPDH were obtained from KangChen Bio (Shanghai, China). Enhanced chemiluminescent (ECL) reagent was supplied by Amersham.

### 2.3 Transthoracic echocardiography

Echocardiography was performed in sedated mice (ketamine, 100 mg/kg, and xylazine, 25 mg/kg, i.p.) using a 30‐MHz probe and the Vevo 3100 Ultrasonograph (VisualSonics) as previously described. The heart rate and body temperature were maintained and recorded. Two‐dimensional directed M‐mode echocardiographic images along the parasternal short axis were recorded to determine left ventricular (LV) size and systolic function. M‐mode measurements included the LV internal dimensions in systole and diastole (LVIDs and LVIDd, respectively) as well as the diastolic thickness LV posterior wall (LVPWd) and the diastolic interventricular septum thickness (IVSd). Per cent fraction shortening was calculated as [(LVIDd − LVIDs)/LVIDd] ×100.

### 2.4 Measurement of cathepsin D and sFlt1 in serum

Cathepsin D and sFlt1 were detected using commercial ELISA kits for cathepsin D and sFlt1 according to the manufacturer's instructions. All samples were simultaneously detected. Serum concentrations of cathepsin D and sFlt1 were determined using standard curves and expressed as units per litre (mg/L). The linear ranges for cathepsin D and sFlt1 were 0‐50 mg/L.

### 2.5 Histological analyses

For histological analyses, mouse hearts were fixed in situ by retrograde perfusion with PBS (pH 7.4) containing 50 mM KCl and 200 U/mL heparin for 2 minutes at 80 mm Hg, followed by in situ paraformaldehyde fixation. Sections were embedded in paraffin and stained with H&E and wheat germ agglutinin (WGA, Alexa Fluor 488 conjugate; Thermo Fisher). Masson (HT15‐1KT; Sigma‐Aldrich) staining was performed to determine collagen deposition following the manufacturer's instruction. Tissue morphometry was performed in a blinded fashion using the Quantimet 500MC digital image analyzer.

### 2.6 Western blotting analysis

The left ventricle tissues were lysed in cell lysis buffer (Beyotime Institute of Biotechnology) at 4°C. Equal amounts of proteins were subjected to 8%‐10% SDS‐PAGE and then transferred onto nitrocellulose membranes (Millipore). The blots were blocked in 10% non‐fat milk in TBST. Membranes were incubated with primary antibodies at 4°C overnight, followed by incubation with secondary antibodies at room temperature for 1 hour. The immunoreactive bands were visualized using ECL kit (Thermo Scientific) and analysed by ImageQuant LAS4000 (GE).

### 2.7 Quantitative real‐time PCR

Total RNA was extracted with TRIzol reagent (Thermo Fisher). Subsequently, 1 μg purified RNA was reversely transcribed into cDNA. qRT‐PCR was performed on an ABI ViiA 7 Real‐Time PCR System (Applied Biosystems) using following primers: VEGFA 5′‐AAGGAGTTCTCTGGTGTGCC‐3′ (forward) and 5′‐CAGGAGGTCGTAGGTCACG‐3′ (reverse); PDGFB 5′‐GCTGAGCGACCACTCCATCC‐3′ (forward) and 5′‐ACTCGGCGATTACAGCAGGC‐3′ (reverse); BFGF 5′‐AAGGACCCCAAGCGGCTCTA‐3′ (forward) and 5′‐CGGTTGGCACACACTCCCTT‐3′ (reverse); GAPDH 5′‐AATCCCATCACCATCTTCCAG‐3′ (forward) and 5′‐AAATGAGCCCCAGCCTTC‐3′ (reverse). GAPDH was used as the housekeeping gene. The relative expression levels of target genes were calculated using the 2^−∆∆^ *^C^* ^T^ method.

### 2.8 Immunohistochemical assessments

Immunohistochemical (IHC) staining was performed using rabbit anti‐CD31 antibody according to the manufacturer's instructions (Leica Bond™ autostainers, Leica Microsystems. Image acquisition was performed using a Nikon eclipse TE2000 inverted microscope.

### 2.9 Statistical analysis

Data were expressed as mean ± SD and analysed by SPSS 18.0 package (SPSS Inc). The obtained data conform the ANOVA assumptions as evaluated using Shapiro‐Wilk normality test and Levene's test for the equality of variances. Comparisons between groups were analysed by two‐way ANOVA with Bonferroni's post‐test. *P* < .05 was considered statistically significant.

## 3 RESULTS

### 3.1 Inhibition of Notch1 induces and promotes postpartum ventricular dilatation

**Figure 1:**
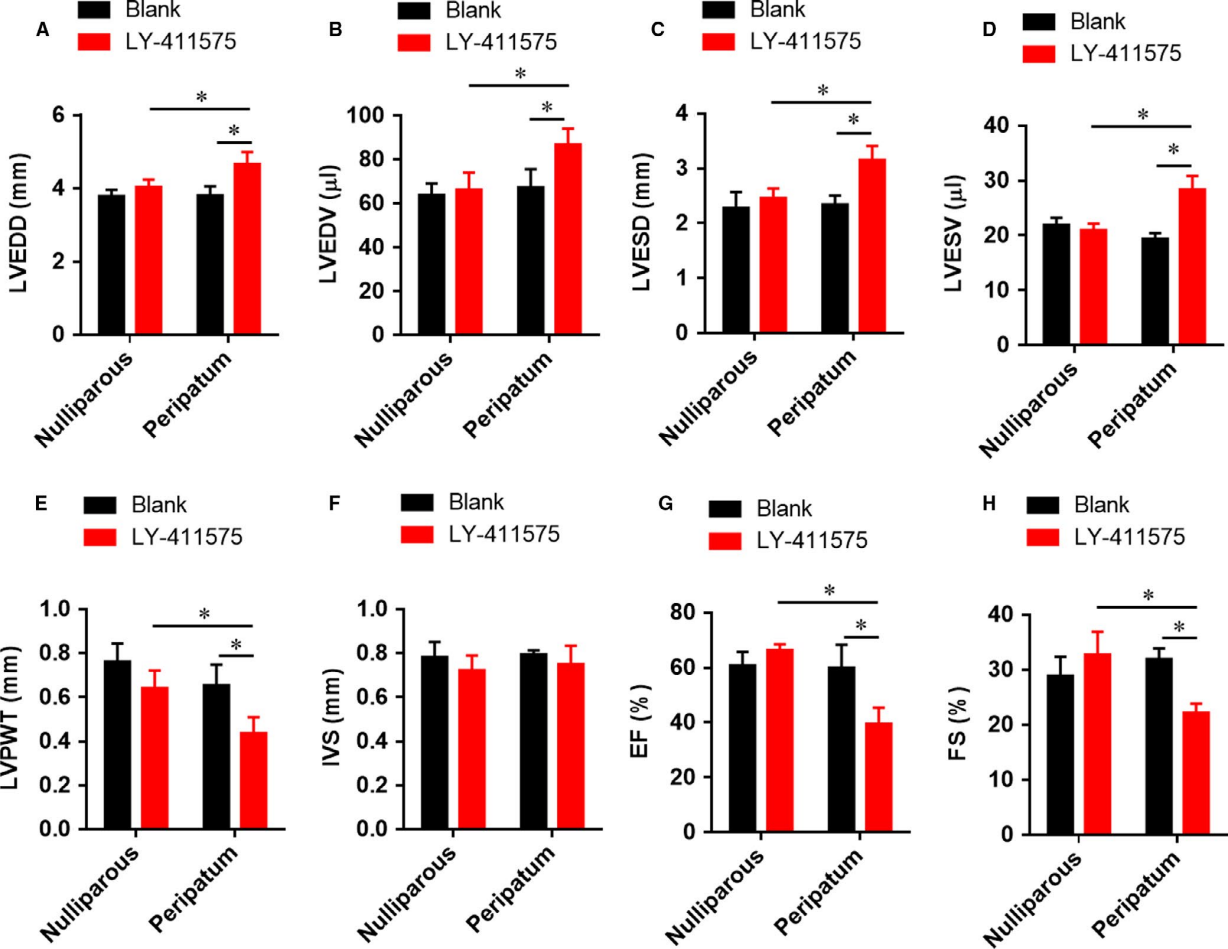
Inhibition of Notch1 induces and promotes postpartum ventricular dilatation. All data were presented as mean ± SD (n = 6). All the parameters were evaluated at 3 weeks post‐partum. A, LVEDD, left ventricular end‐diastolic diameter; B, LVEDV, left ventricular end‐diastolic volume; C, LVESD, left ventricular end‐systolic diameter; D, LVESV, left ventricular end‐systolic volume; E, LVPWT, left ventricular posterior wall thickness; F, IVST, interventricular septal thickness; G, LVEF, left ventricular ejection fraction; and H, LVFS, left ventricular fractional shortening. *P < .05 vs indicated group. Comparisons between groups were analysed by two‐way ANOVA with Bonferroni's post‐test

To explore the role of Notch1 in PPCM, we randomly formed four groups (n = 6) as follows: nulliparous, peripartum, nulliparous*_LY‐411575_* and peripartum*_LY‐411575_*. Figure 1 shows that LVEDD (A), LVEDV (B), LVESD (C), LVESV (D), LVPWT (E), IVST(F), LVEF (G) and LVFS (H) were not significantly changed in peripartum mice without LY‐411575 treatment, indicating these mice did not develop PPCM. However, inhibition of Notch1 by LY‐411575 markedly increased LVEDD, LVEDV, LVESD and LVESV, and decreased LVPWT, LVEF and LVFS, suggesting that blockade of Notch1 led to dilatation of left ventricle and decrease in left ventricular function. Collectively, these data suggested that the Notch1 pathway played a protective role in PPCM, and inhibition of Notch1 could induce and promote PPCM.

### 3.2 Inhibition of Notch1 increases serum cathepsin D and sFlt‐1 and promotes the cleavage of 23‐kD PRL to 16‐kD PRL

**Figure 2:**
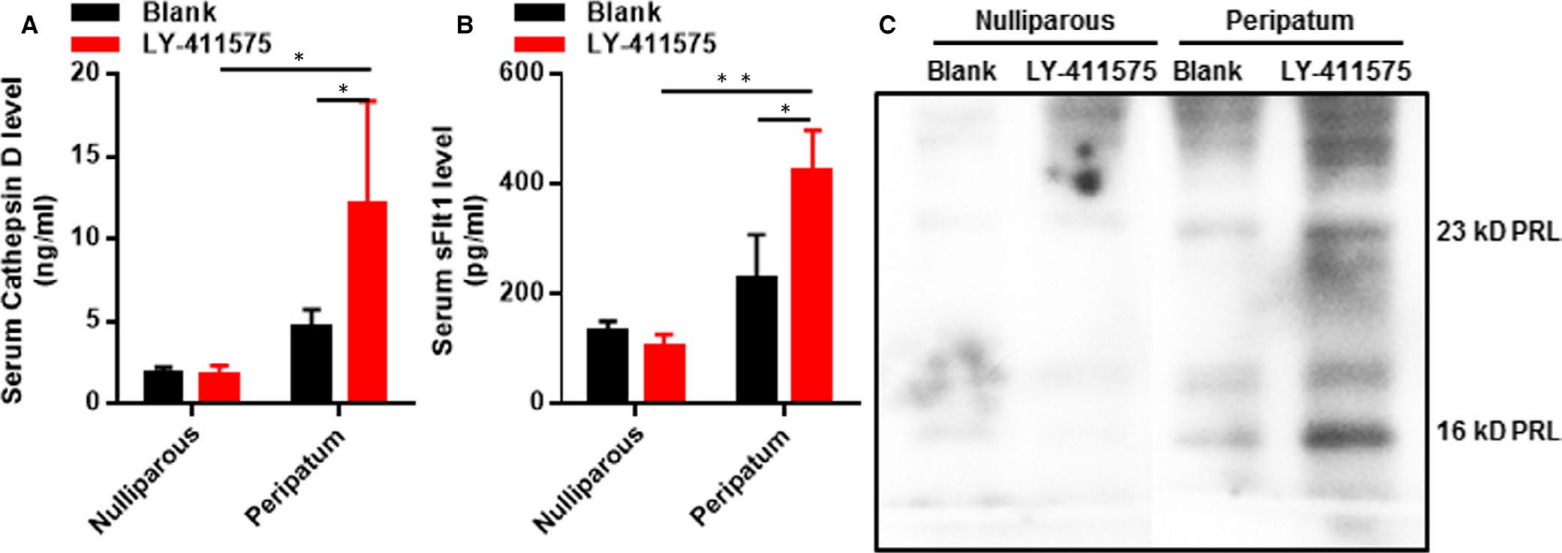
Inhibition of Notch1 increases serum cathepsin D and sFlt‐1 and promote cleavage of 23‐kD PRL to 16‐kD PRL. A, Serum cathepsin D was detected by ELISA. B, Serum sFlt‐1 was detected by ELISA. C, The cleavage of 23‐kD PRL to 16‐kD PRL in serum was determined by Western blot. All data were presented as mean ± SD (n = 6). *P < .05, *P < .05 vs indicated group. Comparisons between groups were analysed by two‐way ANOVA with Bonferroni's post‐test

Since cathepsin D–cleaved 16‐kD PRL and sFlt‐1 have been demonstrated to promote PPCM, and Hes1 has also been suggested to regulate cathepsin D, we wondered whether inhibition of Notch1 affected the expressions of cathepsin D and sFlt‐1 as well as the cleavage of 23‐kD PRL to 16‐kD PRL. Figure 2 shows that serum cathepsin D (Figure 2A) and sFlt‐1 (Figure 2B) levels were just mildly elevated in the peripartum group compared with the nulliparous group. Inhibition of Notch1 by LY‐411575 significantly increased the expressions of cathepsin D and sFlt‐1, and promoted the cleavage of 23‐kD PRL to 16‐kD PRL (Figure 2C). These data indicated that Notch1 could inhibit cathepsin D–mediated PRL cleavage and sFlt‐1 expression.

### 3.3 Inhibition of Notch1 promotes ventricular hypertrophy and myocardial interstitial fibrosis

**Figure 3:**
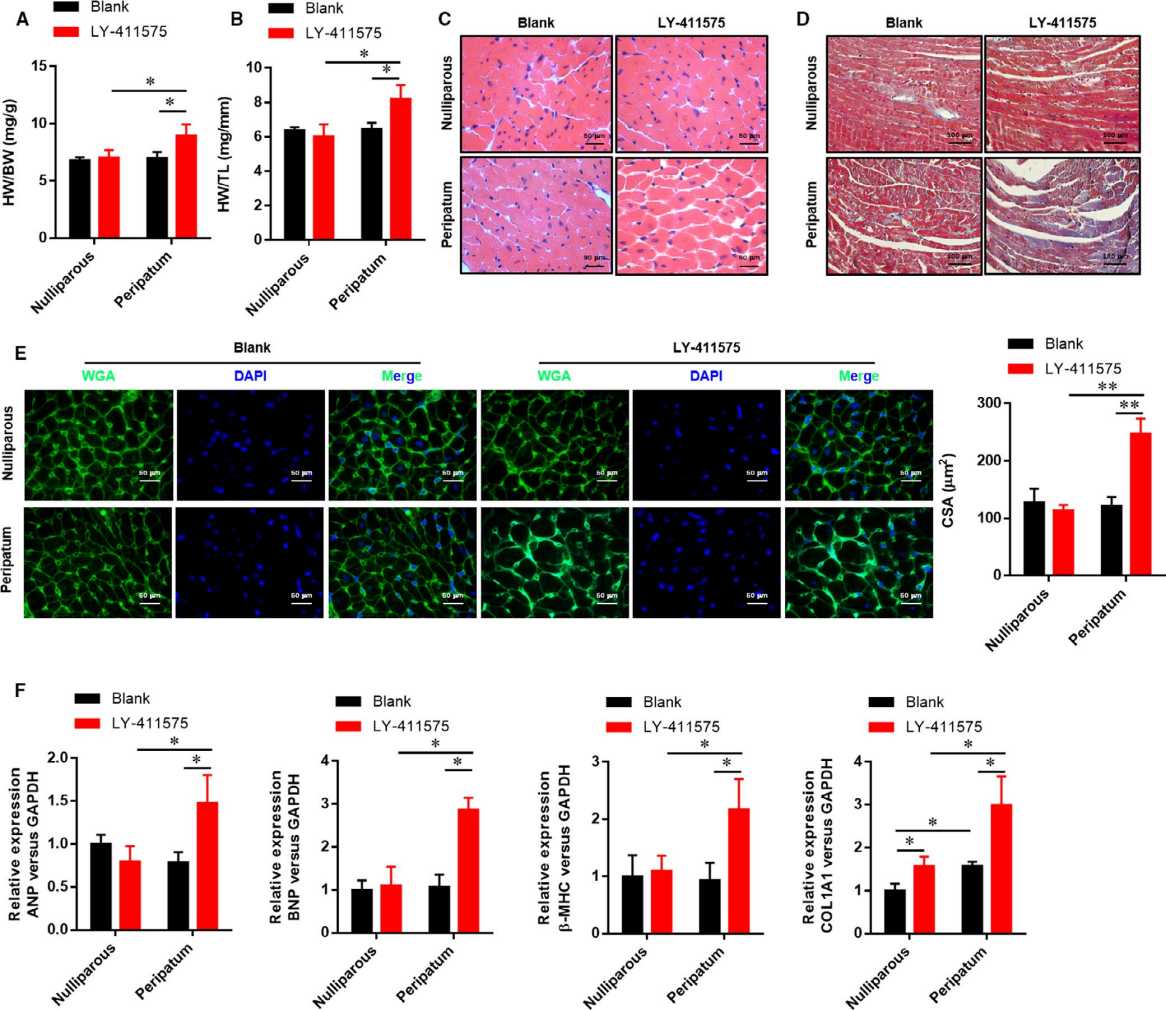
Inhibition of Notch1 promotes myocardial hypertrophy and interstitial fibrosis in the left ventricle. A, The ration of heart weight/bodyweight, HW/BW. B, The ratio of heart weight/tibia length, HW/TL. C, Haematoxylin‐eosin was used to evaluate the ventricular wall thickness and cavity. D, Masson's trichrome staining was used to evaluate the fibrosis. E, Wheat germ agglutinin staining was used to analyse the cardiomyocyte surface area. F, The relative expression of ANP, BNP, β‐MHC and COL1A1 mRNA was evaluated by real‐time PCR. All data were presented as mean ± SD (n = 6). *P < .05, **P < .01 versus indicated group. Comparisons between groups were analysed by two‐way ANOVA with Bonferroni's post‐test

Ventricular hypertrophy and myocardial interstitial fibrosis are remarkable pathological changes in PPCM. To confirm whether Notch1 was involved in these histopathological changes, we compared the HW/BW (Figure 3A) and HW/TL (Figure 3B) among different groups and evaluated histological changes of myocardium (Figure 3C). It demonstrates that there was just interstitial fibrosis (Figure 3D) and mild hypertrophy of cardiomyocytes (Figure 3E) in the peripartum group compared with the nulliparous group. Moreover, we detected the expression of hypertrophic (ANP, BNP and β‐MHC) and fibrotic (COL1A1) genes in the hearts (Figure 3F), which were dramatically elevated in the Notch1 inhibited peripartum group. Inhibition of Notch1 accentuated cardiomyocyte hypertrophy and interstitial fibrosis. Collectively, these data suggested that Notch1 was involved in histopathological changes in PPCM.

### 3.4 Inhibition of Notch1 suppresses postpartum myocardial angiogenesis

**Figure 4:**
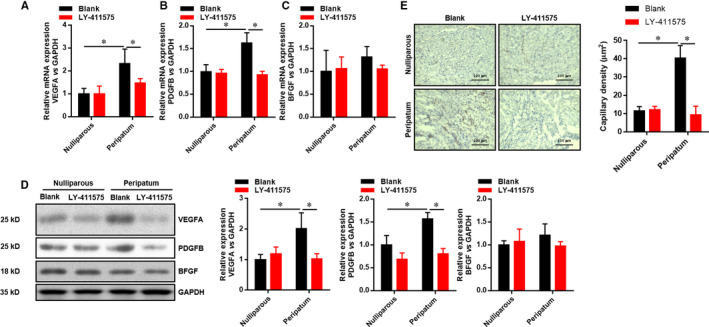
Inhibition of Notch1 suppresses postpartum myocardial angiogenesis. A‐C, The relative expression of VEGFA, PDGFB and BFGF mRNA in LV tissues was evaluated by real‐time PCR. D, The relative expression of VEGFA, PDGFB and BFGF protein in LV tissues was evaluated by Western blot. E, The micro‐vessel density as indicated by CD31 IHC staining. All data were presented as mean ± SD (n = 6). *P < .05 versus indicated group. Comparisons between groups were analysed by two‐way ANOVA with Bonferroni's post‐test

Myocardial angiogenic imbalance is essential for PPCM. Here, we evaluated myocardial capillary density and detected angiogenic factors among different groups. Figure 4 exhibits that there were no significant differences in myocardial capillary density and the expressions of VEGFA, PDGFB and BFGF at the mRNA and protein levels in the peripartum group compared with the nulliparous group. Inhibition of Notch1 markedly decreased the expressions of VEGFA (Figure 4A), PDGFB (Figure 4B) and BFGF (Figure 4C) at the mRNA and protein levels (Figure 4D), and myocardial capillary density (Figure 4E) in the peripartum group. Taken together, these data indicated that inhibition of Notch1 induced and promoted PPCM via suppressing myocardial angiogenesis.

### 3.5 Inhibition of Notch1 decreases N1ICD, Hes1 and p‐Stat3 and increases cathepsin D

**Figure 5:**
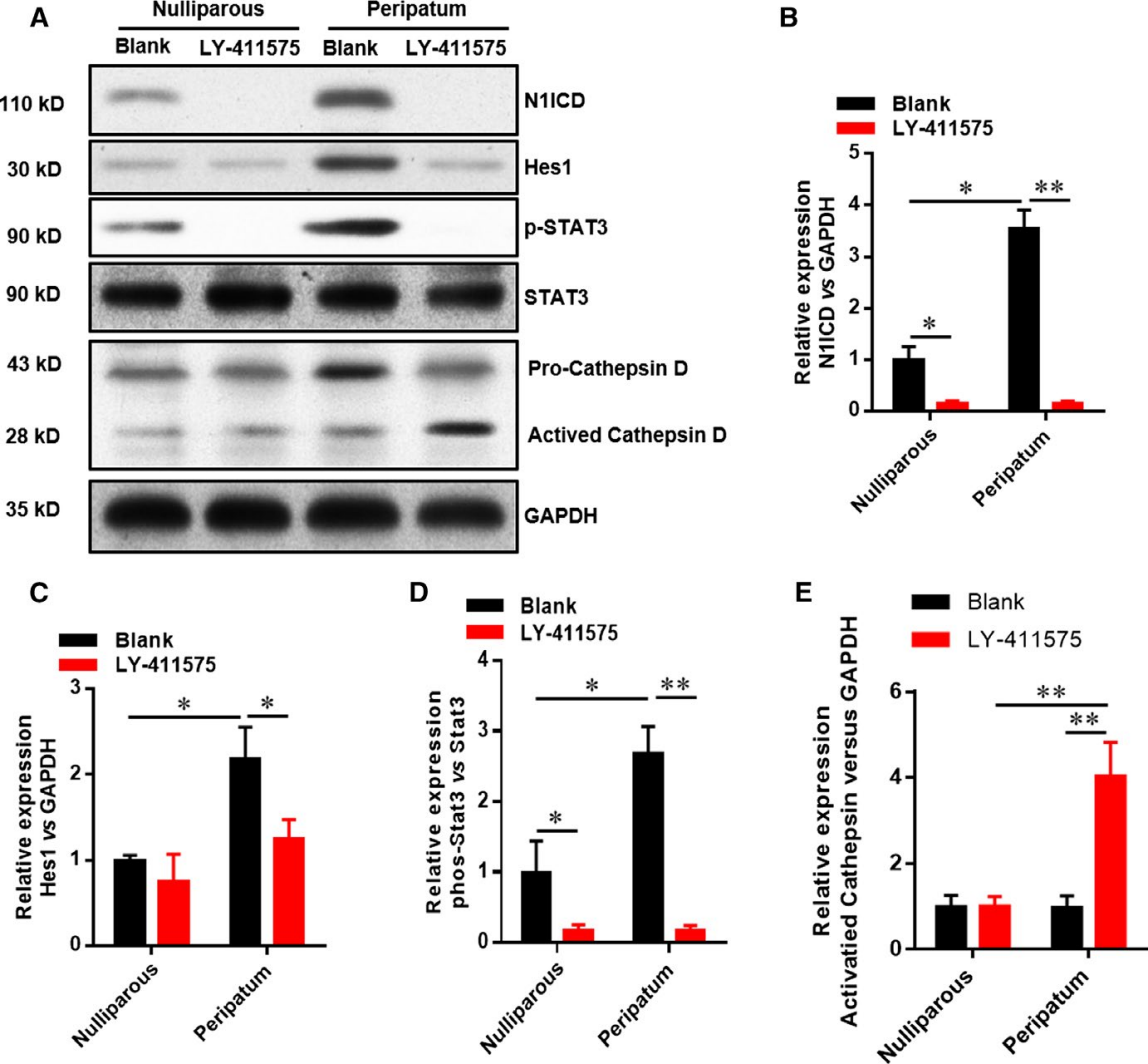
Inhibition of Notch1 decreases N1ICD, Hes1, and p‐Stat3 and increases cathepsin D in left ventricle tissue. All data were presented as mean ± SD (n = 6). The relative expression of N1ICD, Hes1, p‐Stat3 and cathepsin D protein was evaluated by Western blot. *P < .05, *P < .05 vs indicated group. Comparisons between groups were analysed by two‐way ANOVA with Bonferroni's post‐test

The above‐mentioned findings showed that Notch1 was involved in PPCM. As a canonical target gene of Notch1, we wondered whether Hes1 mediated Notch1‐regulated PPCM. Figure 5 displays that the expressions of N1ICD, Hes1, p‐Stat3 and cathepsin D were up‐regulated in the peripartum group compared with the nulliparous group. Inhibition of Notch1 significantly down‐regulated the expressions of N1ICD, Hes1 and cathepsin D, and reduced the phosphorylation level of Stat3. Taken together, the inhibition of Notch1/Hes1 pathway decreased the cathepsin D expression via suppressing Stat3 phosphorylation.

## 4 DISCUSSION

The aetiology and aetiopathogenesis of PPCM remain elusive. However, myocardial hypertrophy, interstitial fibrosis and ventricular dilatation are common pathological changes.^4^ PPCM patients often die of ventricular dysfunction and cardiac failure.^2^ In the present study, pregnant mice in the peripartum group did not show significant LV dilatation, myocardial hypertrophy or interstitial fibrosis, or worsened LV function compared with the nulliparous group. However, inhibition of Notch1 by LY‐411575 markedly exacerbated LV dilatation, myocardial hypertrophy, interstitial fibrosis and LV dysfunction. Within the past decades, there have been salient findings that imbalanced angiogenesis plays an essential role in these pathophysiological changes.^14^

Two pathways, including cathepsin D/PRL and sFlt‐1/VEGF, have been demonstrated to contribute to angiogenic imbalance in PPCM.^2^, ^15^ Hilfiker‐Kleiner and colleagues have found that cathepsin D–cleaved 16‐kD PRL can decrease myocardial capillary density ^2^ and reduce cardiac function, whereas inhibition of PRL secretion by bromocriptine can prevent PPCM and improve LV ejection fraction.^16^ Consistently, Nakajima ^17^ and coworkers have found the expression of 16‐kD PRL and cathepsin D activity in serum of patients with pre‐eclampsia is also markedly elevated, which is a high risk factor of PPCM. Moreover, PRL infusion induces cardiac inflammation.^18^ Stat3 is found to be activated in the maternal heart in pregnancy and postpartum, while it is decreased in PPCM patients, and deletion of Stat3 can initial PPCM, impair myocardial angiogenesis, increase oxidative stress and markedly reduce generation of 16‐kD PRL, indicating a protective role of Stat3 in PPCM.^2^, ^13^ Mechanistically, Stat3 can suppress cathepsin D activity via scavenging reactive oxygen species (ROS).^2^ On the other hand, plasma concentration of anti‐angiogenic sFlt‐1 is significantly higher in PPCM patients and patients with pregnancy‐induced hypertension (PIH)/pre‐eclampsia compared with healthy women, even in recovered PPCM patients.^15^ Actually, higher sFlt‐1 levels are correlated with more severe symptoms and major adverse clinical events.^15^ Conversely, pro‐angiogenic factors, such as VEGF and PIGF, are markedly decreased in patients with PPCM or PIH/preeclampsia. Mechanistically, sFlt‐1 can compete with VEGF receptors (VEGFR1 and VEGFR2) for VEGF and PIGF binding, preventing the interaction between VEGF/PIGF and VEGFR1/2.^19^

Investigators have found that the activation of Notch1 and Hes1 can promote angiogenesis and vascular endothelial cell injury repair.^20^, ^21^ Consistently, our previous studies have also suggested that Notch1 promotes VEGF‐mediated angiogenesis and inhibits myocardial interstitial fibrosis. Zhu's^22^ research has revealed that Nocth1/Hes1 can increase VEGFA expression, thus enhancing angiogenesis. Importantly, our previous research has found that Hes1 can directly enhance the phosphorylation of Stat3 and consequently inhibit cell apoptosis and generation of ROS.^7^ These findings suggest that Notch1/Hes1 pathway participates in the activation of Stat3. According to the study of Ricke‐Hoch,^18^ pregnant mice with a cardiomyocyte‐restricted deletion of Stat3 display cardiac hypertrophy, lower capillary density and increased cathepsin D activity. In addition, cardiac inflammation and fibrosis are also accelerated in Stat3‐depleted mice. Our data here showed that inhibition of Notch1 by LY‐411575 significantly down‐regulated the expressions of N1ICD, Hes1, p‐Stat3 and pro‐angiogenic factors, such as VEGFA, PDGFB and BFGF, and increased the production of cathepsin D and anti‐angiogenic factors, such as sFlt‐1 and 16‐kD PRL. Consistent with these changes, we found significant myocardial hypertrophy and myocardial interstitial fibrosis as well as reduced myocardial capillary density in LY‐411575‐treated mice. Overall, these findings strongly indicated that the sequential activation of Notch1/Hes1/Stat3 might contribute to correcting angiogenic imbalance and alleviating PPCM.

## 5 CONCLUSION

Our data strongly supported the idea that imbalances in angiogenic signalling contribute to PPCM, and Notch1/Hes1pathway may play a protective role in such disorder via regulating cathepsin D and sFlt‐1.

## CONFLICT OF INTEREST

None.

## AUTHOR CONTRIBUTIONS

RR Zhu, ZB Liu and XL Zhou conceived of the study and participated in its design and coordination. RR Zhu performed all experiments. JL Liu, HG Ruan and QC Wu analysed and interpreted the data. The draft was improved through discussion and editing by all the authors, who read and approved the final manuscript.

## Data Availability

The data sets used and analysed during the current study are available from the corresponding author on reasonable request.
